# Modified Team-Based Learning in an Ophthalmology Clerkship in China

**DOI:** 10.1371/journal.pone.0154250

**Published:** 2016-04-21

**Authors:** Zheqian Huang, Miaoling Li, Yuxian Zhou, Yong Ao, Wei Xin, Yu Jia, Ying Yang, Yu Cai, Chaochao Xu, Yangfan Yang, Haotian Lin

**Affiliations:** State Key Laboratory of Ophthalmology, Zhongshan Ophthalmic Center, Sun Yat-sen University, Guangzhou, Guangdong, People´s Republic of China; School of Ophthalmology and Optometry and the Affiliated Eye Hospital, Wenzhou Medical University, CHINA

## Abstract

**Objective:**

Team-based learning (TBL) is an increasingly popular teaching method in medical education. However, TBL hasn’t been well-studied in the ophthalmology clerkship context. This study was to examine the impact of modified TBL in such context and to assess the student evaluations of TBL.

**Methods:**

Ninety-nine students of an 8-year clinical medicine program from Zhongshan Ophthalmic Centre, Sun Yat-sen University, were randomly divided into four sequential units and assigned to six teams with the same faculty. The one-week ophthalmology clerkship module included traditional lectures, gross anatomy and a TBL module. The effects of the TBL module on student performance were measured by the Individual Readiness Assurance Test (IRAT), the Group Readiness Assurance Test (GRAT), the Group Application Problem (GAP) and final examination scores (FESs). Students’ evaluations of TBL were measured by a 16-item questionnaire. IRAT and GRAT scores were compared using a paired t-test. One-way analysis of variance (ANOVA) and subgroup analysis compared the effects among quartiles that were stratified by the Basic Ophthalmology Levels (BOLs). The BOLs were evaluated before the ophthalmology clerkship.

**Results:**

In TBL classes, the GRAT scores were significantly higher than the IRAT scores in both the full example and the BOL-stratified groups. It highlighted the advantages of TBL compared to the individual learning. Quartile-stratified ANOVA comparisons showed significant differences at FES scores (P < 0.01). In terms to IRAT, GRAT and GAP scores, there was no significant result. Moreover, IRAT scores only significantly differed between the first and fourth groups. The FES scores of the first three groups are significantly higher than the fourth group. Gender-specific differences were significant in FES but not the IRAT. Overall, 57.65% of student respondents agreed that TBL was helpful. Male students tended to rate TBL higher than female students.

**Conclusion:**

The application of modified TBL to the ophthalmology clerkship curriculum improved students’ performance and increased students’ engagement and satisfaction. TBL should be further optimized and developed to enhance the educational outcomes among multi-BOLs medical students.

## Introduction

Traditional didactic lecture is the most common method for medical education. It has the inherent advantage of being able to address and provide knowledge to a large group of students in a short period of time. The main drawback of the lecture method is that the audiences receive information passively. Therefore, it may impair the students’ opportunity of critical thinking and long-term knowledge retention.[1] To overcome this drawback, the medical colleges are in the midst of a paradigm shift–moving from a passive, teacher-centred and individual-based way of education, towards a more active, student-centred and group-based way of education.[2]

Team-based learning (TBL) is a teaching method that focuses on the needs of students. The program is directed by a subject specialist. It gives the students opportunities to apply their knowledge through a series of activities. These include individual work, team work, immediate feedback, and task-based problem-solving assignments.[3] TBL has been studied extensively by a great number of medical educators on different audiences (second-year medical students,[4] clerkships[2,5–7] and residents[7,8]), in both short-term and long-term,[6,9,10] and in relations to various educational components (such as attitude,[11] leadership and professionalism[12]). Improved academic outcomes and examination scores have been reported in histology,[13] anatomy[14] and psychiatry.[15] However, there are only limited researches that tried to integrate TBL into ophthalmology clerkship curricula.[16] In this study, we aimed to assess the academic impacts of TBL on ophthalmology clerkships and sought to investigate whether any other effects of TBL would emerge in that context.

## Materials and Methods

### Establishment of Teams

Prior to the module, ninety-nine students of an eight-year clinical medicine program at Sun Yat-sen University were divided into four sequential units with the same faculty. Students of each unit were further assigned to six teams. Each team has three to six students. To prevent students from organizing themselves into pre-existing subgroups (e.g. a unisexual team) based on their preferences, instructors formed the teams randomly with instructions. The teams were given a permanent space in the classroom during the entire module. All the procedures in this study were videotaped, with the approval of the institutional review board of Zhongshan Ophthalmic Centre of Sun Yat-sen University (IRB-ZOC-SYSU). Written informed consents have been obtained from all students.

### Design of the TBL Sessions

The one-week ophthalmology clerkship module included multiple teaching modalities: traditional lectures, gross anatomy, and a TBL module. The TBL module was designed according to the guidance[17] and included: an Individual Readiness Assurance Test (IRAT), preparatory assignments, a Group Readiness Assurance Test (GRAT), a Group Application Problem (GAP) and final examination scores (FESs).

All questionnaires in this study were designed according to the previous TBL studies in other subjects of medical education.[18–20] We have modified it based on the characteristics of ophthalmology. Additional adjustments have been made based on the responses from students. The additional adjustments are relate to the time arrangements. First, the students required sufficient time to prepare for the GAP, considering the time limitation of one-week clerkship study, the time for studying preparatory assignment has been brought forward. Second, due to time restrictions the IRAT and GRAT were reduced to 15 minutes, which the course team felt should be sufficient time for students to participate in further discussion with teammates.

#### Individual Readiness Assurance Test

One week before the clerkship, the students were directed to read a whole text book. Based on self-study prior to the TBL module, each instructional unit began with a 6-question, 15 minutes, closed book, multiple-choice or short answer test on subjects including dry eye, keratitis, cataracts, glaucoma, refractive error, and eye and systemic diseases. The questions in the IRAT measured how well the students understand the important basic ophthalmic concepts and apply the concepts to the practice of medicine. The answers were recorded on paper and submitted for later grading.

#### Preparatory Assignments

After the IRAT, specific GAP assignments were given to the students for preparation. It consisted of creating promotional materials of blindness prevention and treatment of the above-mentioned 6 subjects on the IRAT. The presentations of the GAP assignment were scheduled five days later, providing sufficient time for students to study relevant materials and complete the assignments.

#### Group Readiness Assurance Test

Immediately after completing the preparatory assignments of the GAP, students retook the same IRAT test with a 15-minute time limit. The students were allowed to discuss the test with team members, but they had to submit their answers individually. The results of the GRAT assessed whether a team is more knowledgeable compared with individuals. The answers were also recorded on paper and submitted for later grading.

#### Group Application Problem

In this session, each group had 30 minutes to present their team work. Ten full-time faculty members who participated in the implementation of TBL evaluated the students’ performance on a 10-point scale, which was based on modality, content, ability to improvise, scientific merit and novelty.

### The Student Evaluations

A 16-item questionnaire was conducted at the end of the course to collect students’ self-perceptions on the effects of TBL in the ophthalmology clerkship. Information on the following variables was included: group learning experience (1 item) knowledge acquisition (5 items), motivational dimensions (5 items), instructor performance (1 item), organization (1 item), students’ recommendations (2 items), and overall rating (1 item). Statements were rated on a 6-point Likert scale ranging from “strongly agree” (with the highest score) to “strongly disagree” (with the lowest score). In order to obtain the relation between examination scores and questionnaire responses, data were collected non-anonymously. Subjects emerged from open coding and frequency counts. Two authors (ZQH and MLL) reviewed the answers independently, shared their results to verify their conclusions and arrived at a consensus on issues of disagreement, although one of the authors (ZQH) performed the final review of the feedback.

### Data Collection

The IRAT scores reflected students’ comprehension of content in readings assigned before participating in active learning; the FES reflected practical knowledge and skills gained from the module. In addition, we also implemented the GRAT to assess the students' ability of group problem-solving. The different effects of TBL exerted on the students’ engagement and educational achievement were evaluated based on IRATs, GRATs, FES and the students’ perception of the TBL course. In order to examine whether the TBL course has stronger influences on the students who were more knowledgeable in the subject of ophthalmology, we have divided the students into four quartiles based on their knowledge of Basic Ophthalmology Levels (BOLs). The BOLs are indicated by the students' scores in the most recent ophthalmology exam before the clerkship.

The TBL activity of GAP comprised 5% of the module grade, whereas final examinations contributed the remaining 95%. Students were required to score at least a 70% average on the final exams (without the TBL component) as well as earn a 70% overall module grade (including the TBL component) to pass the module.

### Data Analysis

The IRAT and GRAT scores were compared by a paired t-test. Gender differences in test performance and questionnaire were compared by a t-test. A one-way analysis of variance (ANOVA) was used to compare the IRAT, GRAT, application exercise and final examination scores among the four quartiles of students stratified according to BOLs. All analyses were performed by SPSS software version 22.0 (SPSS Inc., Chicago, IL, USA).

## Results

### Class Performance

**Fig 1:**
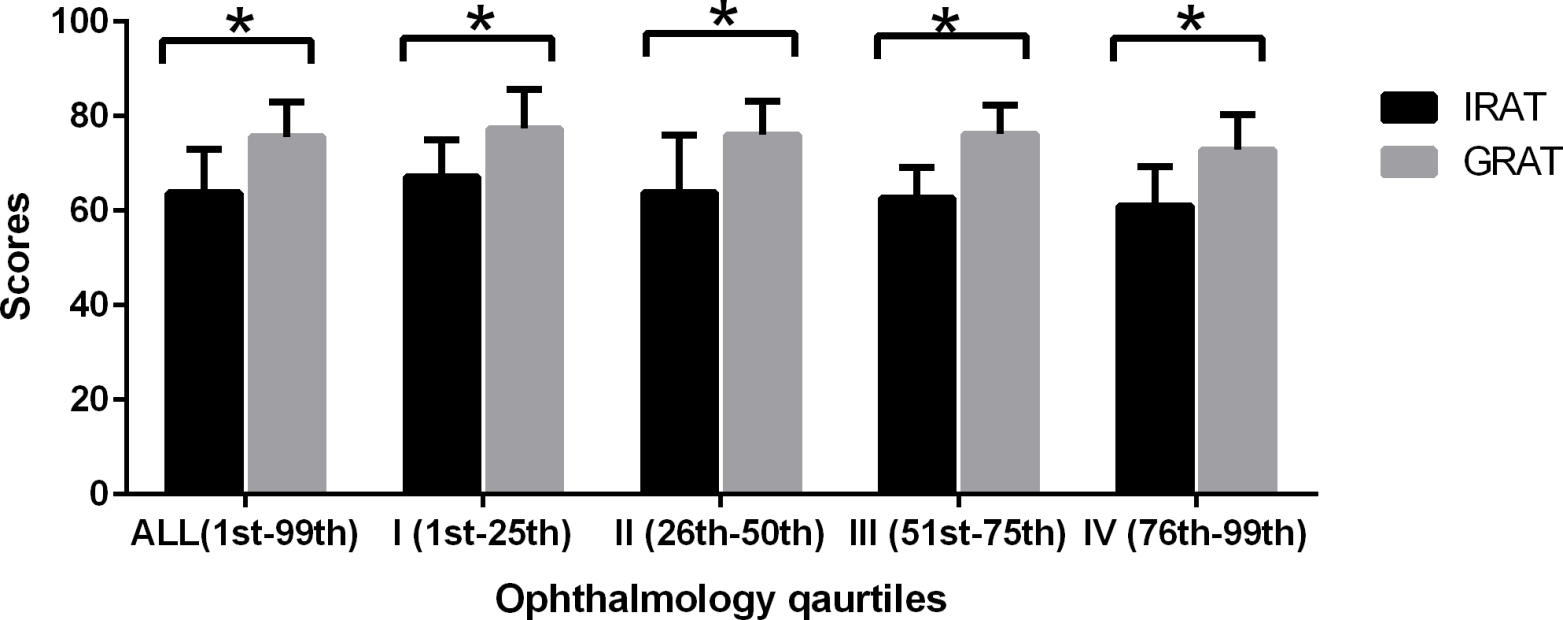
The effect of group learning on students’ performance: comparison of the IRAT and GRAT scores according to BOLs of the most recent ophthalmology exam before the clerkship. By the paired t-test. *The mean difference is significant at the 0.05 level. Individual Readiness Assurance Test (IRAT), Group Readiness Assurance Test (GRAT), Group Application Problem (GAP).

In the TBL classes, the GRAT scores were significantly higher than the IRAT scores, both overall and in the groups stratified by BOLs (Fig 1). These results confirmed that group problem-solving was more effective than individual problem-solving, regardless of BOLs. The ANOVA was used to compare examination scores across ophthalmology levels (Table 1). The IRAT, GRAT and application exercise scores did not differ significantly (P = 0.121, 0.175 and 0.485, respectively), whereas the final examination score revealed significant differences (P = 0.01). When the groups stratified by BOLs are compared with one another, the IRAT scores differed significantly between the first and fourth groups, but not between the second and third groups. By contrast, the third and second groups scored significantly higher than the fourth group with regard to FESs, which showed that TBL improved the performance of academically weaker–but not the weakest–students.

**Table 1:** The impacts of team based learning (TBL) on students’ performance according to BOLs of the most recent ophthalmology exam before the clerkship.

Ophthalmology quartiles	IRAT	GRAT	GAP	FES
ALL(1st–99th)	63.78±9.30	75.65±7.40	4.247±0.45	76.77±4.16
I (1st–25th)	67.22±7.78	77.40±8.26	4.32±0.48	78.72±3.41
II (26th–50th)	63.82±12.18	76.04±7.07	4.21±0.39	77.45±3.39
III (51st–75th)	62.74±6.40	76.19±6.22	4.14±0.53	76.65±4.03
IV (76th–99th)	61.04±8.23	72.88±7.46	4.31±0.44	74.03±4.59
F	1.986	1.689	0.822	6.447
p-value	0.121	0.175	0.485	0.01*
Post-hoc	I>IV p = 0.020	I >IV p = 0.033		I >IV p = 0.000
				II>IV p = 0.002
				III>IV p = 0.025

By one-way ANOVA.

*The mean difference is significant at the 0.05 level. Individual Readiness Assurance Test (IRAT), Group Readiness Assurance Test (GRAT), Group Application Problem (GAP).

**Fig 2:**
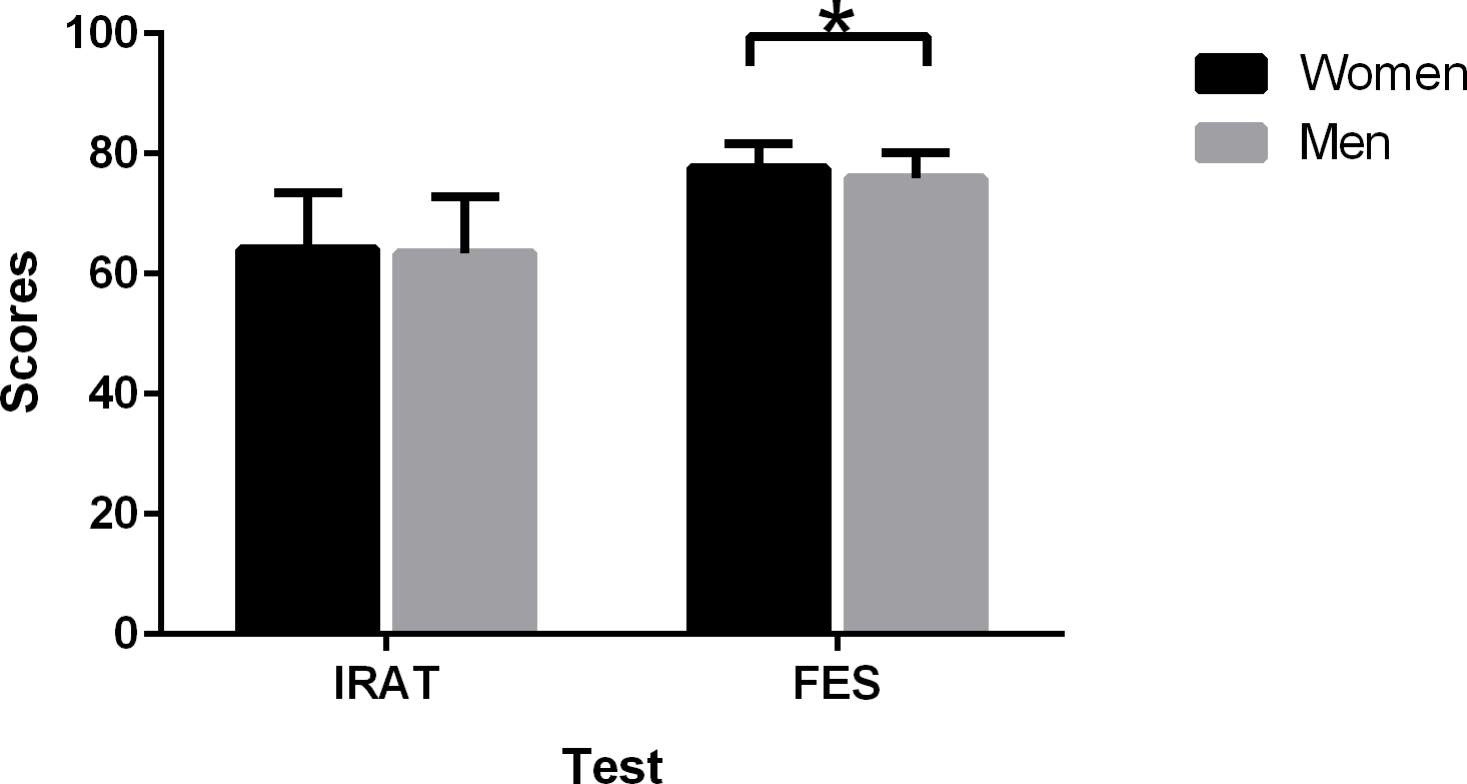
Sex differences in test performance of team-based learning (TBL) students. T-test for differences of means.*The mean difference is significant at the 0.05 level.

There were sex-specific differences in students’ test performances (Fig 2). Female students scored the same as male students on the IRAT; however, on the final exam, female students scored significantly higher than their male peers (P = 0.039; t-test).

### Student Evaluation of TBL

Students were asked to rate their TBL experience at the end of the module (Table 2); 57.65% of students agreed that TBL helped their learning experience. The dissection lab, traditional lectures and textbooks were rated higher. TBL sessions were deemed more helpful than on-line material and educational computer programs by a considerable margin. In a focused evaluation of TBL, students believed TBL helped to assess their present knowledge, provided them with a higher level of knowledge and had a positive impact on their learning attitudes. In addition, 80% of students agreed that the TBL module was well organized and 56.47% thought TBL should be offered more frequently in the curriculum. However, some students felt that the amount of time given to complete the TBL assignment was inadequate in light of other required and on-going learning activities.

**Table 2:** Students’ questionnaire responses to team-based learning (TBL) courses: comparisons between gender and quartile students (1 –strongly disagree, 6 –strongly agree)(85 students).

Questionnaire Item	Percentage responses(n = 85)				Mean score ±standard deviation			Mean score ±standard deviation			
	6–5	4–3	2–1	Total (85)	Women(41)	Man(44)	P	Total (85)	II III(64)	IV(21)	P
TBL helped to assess present knowledge	60.00	35.29	4.71	4.71±1.27	4.39±1.30	5.00±1.18	0.026*	4.71±1.27	4.55±1.32	5.19±0.98	0.043*
TBL helped me to obtain a higher level of knowledge	62.35	36.47	1.18	4.82±1.03	4.54±1.05	5.09±0.94	0.012*	4.82±1.03	4.70±1.05	5.19±0.87	0.058
TBL reduced the amount of time needed for self-study	50.59	30.59	29.41	3.84±1.71	3.49±1.72	4.16±1.66	0.07	3.84±1.71	3.66±1.68	4.38±1.72	0.092
TBL challenged me to give my best	51.76	41.18	7.06	4.53±1.29	4.07±1.37	4.96±1.06	0.001*	4.53±1.29	4.33±1.35	5.14±0.85	0.011*
TBL had a positive impact on my learning attitudes	60.00	36.47	3.53	4.72±1.27	4.42±1.38	5.00±1.10	0.033*	4.72±1.27	4.56±1.28	5.19±1.12	0.048*
TBL is an effective, motivating learning process	58.82	34.12	7.06	4.72±1.25	4.44±1.32	4.98±1.13	0.047*	4.72±1.25	4.58±1.29	5.14±1.01	0.072
The instructor highly facilitated the learning process	58.82	38.82	2.35	4.76±1.16	4.61±1.26	4.91±1.05	0.237	4.76±1.16	4.61±1.16	5.24±1.04	0.030*
The TBL module well organized	80.00	20.00	0.00	5.16±0.88	5.02±0.96	5.30±0.79	0.159	5.16±0.88	5.11±0.94	5.33±0.66	0.317
I will recommend TBL to other students	44.71	45.88	9.41	4.33±1.41	4.07±1.39	4.57±1.40	0.106	4.33±1.41	4.22±1.46	4.67±1.20	0.208
TBL should be offered more frequently in the curriculum	56.47	32.94	10.59	4.47±1.44	4.15±1.51	4.77±1.33	0.045*	4.47±1.44	4.30±1.52	5.00±1.05	0.052
Overall, I am very satisfied with this TBL approach	52.94	38.82	8.24	4.44±1.32	4.20±1.27	4.66±1.35	0.106	4.44±1.32	4.25±1.37	5.00±1.00	0.023*
I frequently study with colleagues	31.76	44.71	23.53	3.81±1.61	3.78±1.56	3.84±1.68	0.864	3.81±1.61	3.61±1.69	4.43±1.21	0.043*
TBL promoted the learning of essential concepts or skills	65.88	27.06	7.06	4.68±1.37	4.49±1.43	4.86±1.30	0.209	4.68±1.37	4.61±1.30	4.91±1.58	0.396
TBL promoted effective cooperative learning	68.24	23.53	8.24	4.84±1.34	4.66±1.46	5.00±1.22	0.244	4.84±1.34	4.75±1.26	5.10±1.58	0.310
TBL promoted increased reading of the textbook by the students	74.12	22.35	3.53	5.07±1.13	4.81±1.29	5.32±0.91	0.036*	5.07±1.13	5.10±1.05	5.00±1.38	0.744
**The following were helpful to my learning:**	
Lectures	78.82	8.82	1.18	5.09±0.91	5.15±0.82	5.05±0.99	0.612	5.09±0.91	5.08±0.88	5.14±1.01	0.779
Dissection labs	92.94	7.06	0.00	5.61±0.62	5.66±0.57	5.57±0.66	0.505	5.61±0.62	5.63±0.63	5.57±0.60	0.733
TBL sessions	57.65	34.12	8.24	4.58±1.27	4.29±1.38	4.84±1.10	0.045*	4.58±1.27	4.48±1.26	4.86±1.28	0.244
Textbooks	62.35	34.12	3.53	4.78±1.13	4.98±1.01	4.59±1.21	0.116	4.78±1.13	4.75±1.08	4.86±1.28	0.708
On-line materials	55.29	38.82	5.88	4.54±1.18	4.59±1.34	4.50±1.02	0.741	4.54±1.18	4.48±1.14	4.71±1.31	0.442
Computer programs	41.18	42.35	16.47	4.07±1.40	4.00±1.47	4.14±1.34	0.655	4.07±1.40	3.92±1.41	4.52±1.29	0.086

T-test for differences of means.

*The mean difference is significant at the 0.05 level.

Notably, there was a sex-specific difference in the evaluation profile. Male students tended to rate most items higher than female students (Table 2). The item “TBL challenged me to give my best” and “TBL had a positive impact on my learning attitudes” received significantly higher mean scores (P = 0.001 and P = 0.033, respectively) from male students than from female students. Male students also rated other general statements higher.

## Discussion

This study assessed the educational effectiveness of TBL in the ophthalmology clerkship context at the Zhongshan Ophthalmic Centre of Sun Yat-sen University. Our results suggested that applying TBL had a positive impact on acquiring ophthalmology knowledge. By working together, students’ GRAT scores were significantly higher than their IRAT scores. Students in the middle academic quartiles (groups II and III) have improved their academic performance. Additionally, the students were satisfied with our TBL approach.

It has been generally accepted that with peer teaching and group learning methods such as TBL, the groups should outperform the individuals. The analysis of readiness assurance tests (Fig 1) indicated that the average group scores were 11% higher than the average individual scores. This result suggested that the interactions occurred in small groups are effective to some extents. During the discussion process, the students could deepen their understanding through communication and debate. The knowledge acquired from exploration is more stable and durable compared to that acquired from listening to teachers. However, we also found that the overall team score did not always surpass the score of the team’s best member. We thought this phenomenon is because that the students in different academic quartiles would share and discuss their ideas with each other, sometimes they might arrive at an incorrect answer which was supported by the majority. However, it could also be due to the fact that one week is too short to develop team accountability.

Previous studies [4,14,15,21] have shown that one of the major benefits of TBL was to improve the middle academic quartile. We evaluated the effect of TBL on students’ performance by dividing students into different groups based on their BOLs to examine the mechanisms underlying this benefit (Fig 1 and Table 1). The result showed that the FESs of the middle academic quartile (groups II, III) had been greatly improved from the IRATs. This might result from the enhancement of personal knowledge through interaction with team members and faculty and the advance assignment. However, there was no evidence showing that helping other students would influence the performance of the students in the highest quartile. In line with the previous study,[22] the final examination score of the lowest academic quartile was significantly lower than that of the other quartiles. We believe it is due to the limited implementation period being too short for the academically weakest students to develop effective team communication and team learning skills. Moreover, TBL is superior to the lecture-based learning only when students have reached a comparably high level of related ophthalmology theories; otherwise, it will be difficult for students to engage in effective discussion without understanding of the abstract ophthalmic concepts.[5]

Our study indicated that the female and male students responded differently to both educational format and knowledge tests (Fig 2). Females outscored males by only 0.7 on the IRAT, but scored 4.3 points higher on the final exam. The TBL seems to be more suitable to female students. Generally speaking, the students are highly satisfied with TBL and their improvements during the experimental period. However, males were significantly more satisfied with TBL than females. These results indicate that the male medical students have higher probability of over-estimating their learning outcomes compared to female students. A previous study indicated that despite performing equally as well as their male peers, female medical students reported decreased self-confidence and increased anxiety.[23] The female students may consistently under estimate themselves because they consider the negative effect of overestimation outweighing that of underestimation. Social norms regarding modesty may weigh more heavily the female students.[24]

The second goal of this investigation was to document students’ evaluations of TBL. These data are somewhat compromised in that only 85 out of 99 (86%) potential participants have completed the survey. The students’ evaluations (Table 2) indicated that the TBL is an effective learning process for ophthalmology. More than half of the students reported that the TBL helped them assess their present knowledge, achieve a higher level of knowledge, and reduce the amount of time required for self-study, in addition to having a positive impact on their learning attitudes. Overall, students were satisfied with the TBL module. They also commended that it should be used more frequently in medical education.

On the other hand, most students still considered traditional didactic instruction and textbooks to be more appropriate than the TBL module, because the majority of students typically used to study alone. The questionnaire revealed that only one-third of the students studied with colleagues frequently. Many students felt uncomfortable with team learning and had never discovered its value. These students might rate TBL based on how much information acquired from the course can actually be applied in further examinations. The scores in exams have been considered as the most important assessed outcome.[25] On the basis of these observations, we propose that students with who achieve higher performance are more prepared to overcome any negative implications of TBL and can use interactive learning successfully in mastering their ophthalmology studies.

This study has several limitations. First, we have modified TBL by replacing peer evaluation with faculty evaluation which was based on the student’s participation and performance during the module. While faculty evaluation may be more objective, it is likely to reduce accountability to the team not using peer evaluation. Second, the relatively small sample size limited the study’s statistical power and prevented statistically significant results. The response rate for the evaluations of the TBL was low (85 out of 99), which may have biased our results. Third, the one-week ophthalmology clerkship does not provide enough time for the students to develop high-functioning learning teams, which may reduce the effects of TBL. Because the final examination was conducted right after the TBL module, our results only revealed a relatively short-term impact on learning. Fourth, our study design did not allow us to compare the students’ performance among the different teaching techniques (i.e., PBL, TBL and classical case-based small group discussions). Our results are encouraging, but more outcome-centred studies of TBL are required to provide objective evidence of the effectiveness of this active learning strategy in medical education. Further study can explore the long-term effects of TBL strategies in the ophthalmology clerkship curriculum. Additionally, a prospective research design that compares the learning outcomes of academically similar student cohorts exposed to the TBL strategy versus another active learning method might produce meaningful data. We expect that satisfaction with team experience would increase year after year as the faculty and students become familiar with TBL and ultimately benefit from it.

In conclusion, the application of TBL to the ophthalmology clerkship curriculum improved students’ performance, particularly that of academically weaker students–and increased students’ engagement and satisfaction. We believe that with time and experience, TBL will prove to be an effective and highly rated innovative learning method in ophthalmology, leading to more faculty members to adopt it in their teaching and providing more active learning and deeper understanding for medical students.
